# Prevalence and Associated Factors of Passive Smoking among Women in Jilin Province, China: A Cross-Sectional Study

**DOI:** 10.3390/ijerph121113970

**Published:** 2015-10-30

**Authors:** Zhijun Li, Yan Yao, Yaqin Yu, Jieping Shi, Yawen Liu, Yuchun Tao, Changgui Kou, Huiping Zhang, Weiqing Han, Yutian Yin, Lingling Jiang, Bo Li

**Affiliations:** 1 Department of Epidemiology and Biostatistics, School of Public Health, Jilin University, Changchun 130021, China; E-Mails: beyond.hehe@163.com (Z.L.); yaoyan@jlu.edu.cn (Y.Y.); yuyaqin5540@163.com (Y.Y.); mary_shijp@163.com (J.S.); ywliu@jlu.edu.cn (Y.L.); taoyuchun@163.com (Y.T.); koucg@jlu.edu.cn (C.K.); hanwq1987@foxmail.com (W.H.); yinyutian1123@126.com (Y.Y.); jianglingling.2008@163.com (L.J.); 2 Department of Psychiatry, School of Medicine, Yale University, New Haven, CT 06511, USA; E-Mail: huiping.zhang@yale.edu

**Keywords:** passive smoking, prevalence, socio-demographic factors, adult females

## Abstract

*Background:* The present study aimed to investigate the prevalence and associated socio-demographic factors of passive smoking among women in Jilin Province, China. *Methods:* A cross-sectional study was conducted in 2012, using a self-reported questionnaire interview. A representative sample of 9788 non-smoking women aged 18–79 years was collected in Jilin Province of China by a multistage stratified random cluster sampling design. Descriptive data analysis and 95% confidence intervals (CI) of prevalence/frequency were conducted. Multivariable logistic regressions were used to examine the associated socio-demographic factors of passive smoking. *Results:* The overall prevalence of passive smoking among non-smoking women in Jilin Province was 60.6% (95% CI: 59.3–61.8), 58.3% (95% CI: 56.7–59.9) from urban areas, and 63.4% (95% CI: 61.6–65.3) from rural areas. Twenty-six percent (95% CI: 24.9–27.1) of the non-smoking women reported daily passive smoking, of which 42.9% (95% CI: 41.6–44.1) reported passive smoking at home, and 5.1% (95% CI: 4.5–5.7) reported passive smoking in restaurants. Women in urban areas were less likely to be passive smokers than those in rural ones (OR-Odds Ratio: 0.825, 95% CI: 0.729–0.935), elderly women were less likely to be passive smokers than younger women (55–64 years OR: 0.481, 95% CI: 0.342–0.674; 65–79 years OR: 0.351, 95% CI: 0.241–0.511). Seperated/divorced women were less likely to be passive smokers (OR: 0.701, 95% CI: 0.500–0.982), and widowed women (OR: 0.564, 95%CI: 0.440–0.722), as the married were the reference group. Retired women second-hand smoked due to environmental causes significantly less than manual workers (OR: 0.810, 95% CI: 0.708–0.928). Women with a monthly family income of more than 5000 RMB were less likely to be passive smokers than those with an income less than 500 RMB (OR: 0.615, 95% CI: 0.432–0.876). *Conclusions:* The prevalence of passive smoking is lower than that reported in 2010 Global Adult Tobacco Survey (GATS) China, but passive smoking is still prevalent and has been an acute public health problem among non-smoking women in Jilin Province, China. Our findings suggest an urgent need for tobacco control and the efforts of public health should be both comprehensive and focus on high-risk populations in Jilin Province, China.

## 1. Introduction

Smoking and passive smoking are a major global public health problem, a major influence factor of chronic diseases and neonatal death, and the major global preventable risk factors of death [1,2,3]. There are more than 1.1 billion smokers around the world, and the number of smokers is increasing sharply at a rate of 2% per year [4]. World Health Organization reported that tobacco use contributed to more than five million deaths a year; if no efficient measures towards tobacco control are enacted, the deaths by tobacco use will increase to 10 million, and 80% of them will be concentrated in developing countries [4]. China is the world’s largest producer and consumer of tobacco products [5]. The 2002 and 2010 national survey suggested that the prevalence of smoking has been declining, but passive smoking status is still serious: 51.9% of non-smokers were regularly exposed to passive smoking in 2002 and 72.4% in 2010 [6,7]. Previous studies [8,9,10] have shown that passive smoking can increase the risk of cancers, cardiovascular disease, chronic obstructive pulmonary disease and asthma, and damage lung function, and is especially detrimental to the health of women and children. More than 10 million deaths are caused by passive smoking annually, according to a 2002 national survey, and women are the major victims, with more than 90% of women exposed at home [6]. Passive smoking is highly prevalent among women and has been a major concern in China.

Multivariable evidence showed that passive smoking is associated with socio-demographic factors [11,12,13,14]. People with a low socio-demographic status are more likely to smoke and the inverse association between educational status and passive smoking has been confirmed in Asia areas [12,15,16]. However, previous studies have mostly focused on male or population data [17,18]; thus few data are available concerning the relationship between passive smoking and socio-demographic factors among non-smoking women in Jilin Province, China. It is critical that we understand the socio-demographic factors associated with passive smoking among non-smoking women; only through understanding these risk factors can we hope to enact effective tobacco control policies to protect the population. The present study aimed to investigate the prevalence and associated socio-demographic factors of passive smoking among women in Jilin Province, China.

## 2. Experimental Section

The study data were acquired from the Project on Present Situation and Change Forecast of Disease Spectrum in Jilin Province of China. The investigation was a population-based cross-sectional survey. Multistage stratified cluster sampling method was used to select the study sample of populations aged 18–79 years and who had lived in Jilin Province, China for at least six months. The multistage stratified cluster sampling method was used to select the study sample. Nine regions (Changchun, Jilin, Siping, Liaoyuan, Tonghua, Baishan, Songyuan, Baicheng, and Yanbian), 32 districts or counties, 95 town or communities, and 45 units in Jilin Province were selected. Lastly, all adult resident were selected from each household of the selected town or communities. The detailed stratifying process was reported previously [19]. 23,050 Subjects aged over 18 years were recruited. 21,435 Subjects completed the survey, resulting in a response rate of 84.9%. Response rates of urban and rural areas were 81.8% and 88.6%, respectively. A total of 9788 non-smoking women were chosen by the study. Ethical approval was obtained by Jilin University School of Public Health, and written informed consent was obtained from all subjects.

All interviews were conducted by trained investigators. The questionnaire included information on socio-demographic characteristics and passive smoking status. “Passive smoking” was defined as those who perceived to be or were exposed to second-hand smoke or a smoking environment from smokers during past seven days.

All data analyses were weighted to make the sample representative of the population in Jilin Province by post-stratification adjustment according to the following factors: region, urban/rural, age, and gender groups, according to the 2010 China (Jilin Province) Population Census. Descriptive data analysis and 95% confidence intervals (CI) of prevalence/frequency were conducted. Rao-Scott Chi-square tests were used to compare the prevalence of passive smoking in different groups. In order to adjust for potential confounding effects, multiple logistic regression analyses were carried out to find the independent factors associated with passive smoking. In the regression model, seven covariates were included to study the associations between socioeconomic characteristics towards tobacco control with passive smoking among non-smoking females. The Cox and Snell test was used to evaluate the overall model performance, which performed steadily (Cox and Snell test: *p* = 0.259). All data were analyzed by the complex sampling function of SPSS 22.0 (IBM Corp, Armonk, NY, USA), and *p* ≤ 0.05 was considered to be statistically significant.

## 3. Results

The sample included 9788 non-smoking females, representative of the general Jilin Province non-smoking women aged 18 years and over by socio-economic characteristics (Table 1). In the study, the mean age was 47.24 ± 12.88 years, 56.2% from the urban area, and 91.8% were Han Chinese. The majority of the subjects were between 35–54 years of age, 23.8% were aged between 35–44; 74.3% attained an education level junior middle school or higher; 81.0% were married; 46.4% were manual workers; and 35.4% had a family monthly income between 1000 and 2000 RMB.

**Table 1:** Socio-demographic characteristics among non-smoking women aged 18 years or over in Jilin Province, China.

Characteristics	*n*	%
**Region**		
Urban	5232	56.2
Rural	4556	43.8
**Ethnic**		
Han	9030	91.8
Minorities	758	8.2
**Age**		
18–24	447	15.0
25–34	1218	18.9
35–44	2465	23.8
45–54	2720	20.4
55–64	2018	13.7
65–79	920	8.1
**Education**		
Primary school and below	3126	25.7
Junior middle school	2720	29.4
Senior middle school	2398	24.9
College and above	1544	20.1
**Marital status**		
Married	8470	81.0
Single	517	12.4
Separated/Divorced	159	1.5
Widowed	642	5.1
**Occupation**		
Manual	4768	46.4
Skilled	1982	23.6
Retired	3038	29.9
**Family monthly income**		
<500	2048	17.4
500–999	1921	19.1
1000–1999	3116	35.4
2000–2999	1483	18.0
3000–4999	635	7.8
≥5000	197	2.3

Note: Complex weighted computation was used in the statistical analysis.

The percent distribution of passive smoking of Jilin Province women aged 18 years or over in 2012 is given in Table 2. Overall, 60.6% (95% CI: 59.3–61.8) of Jilin Province non-smoking women described themselves as passive smokers (58.3% of urban and 63.4% of rural). During the past seven days, 26.0% (95% CI: 24.9–27.1) of the non-smoking women reported daily passive smoking exposure, 13.9% (95% CI: 13.1–14.8) were passive smokers more than three days per week, and 20.3% (95% CI: 19.3–21.4) less than three days per week. The frequency distributions of passive smoking in urban were higher than that in rural. Approximately 42.9% (95% CI: 41.6–44.1) of passive smoking exposure was at home, 15.2% (95% CI: 14.3–16.2) at workplaces, and 8.6% (95% CI: 7.9–9.3) in social environments. However, the proportion report of passive smoking in restaurant was lower (5.1%) (95% CI: 4.5–5.7).

**Table 2:** Percent distribution and corresponding 95% confidence intervals (CI) of passive smoking among non-smoking women aged 18 years or over in Jilin Province, China.

Characteristics	Urban (*n* = 5232) % (95% CI) (*n*)	Rural (*n* = 4556) % (95% CI) (*n*)	Total (*n* = 9788) % (95% CI) (*n*)
**Passive smoking**	58.3 (56.7–59.9) (2976)	63.4 (61.6–65.3) (2731)	60.6 (59.3–61.8) (5707)
**Frequency per week**			
Everyday	21.8 (20.5–23.1) (1171)	31.3 (29.5–33.2) (1386)	26.0 (24.9–27.1) (2557)
≥3 d/week	14.0 (12.9–15.2) (729)	13.8 (12.6–15.2) (617)	13.9 (13.1–14.8) (1346)
1–3 d/week	22.3 (20.9–23.7) (1076)	17.7 (16.2–19.4) (728)	20.3 (19.3–21.4) (1804)
**Sources of Passive smoking**			
Home	37.9 (36.3–39.4) (2004)	49.3 (47.3–51.2) (2198)	42.9 (41.6–44.1) (4202)
Workplace	18.5 (17.3–19.8) (894)	11.0 (9.8–12.4) (411)	15.2 (14.3–16.2) (1305)
Restaurant	6.5 (5.7–7.3) (304)	3.3 (2.6–4.3) (113)	5.1 (4.5–5.7) (417)
Entertainment places	8.9 (8.0–10.0) (414)	8.1 (7.1–9.3) (346)	8.6 (7.9–9.3) (760)
Other	2.2 (1.8–2.7) (113)	2.1 (1.7–2.7) (92)	2.1 (1.8–2.5) (205)

Note: Complex weighted computation was used in the statistical analysis.

Table 3 describes prevalence of passive smoking among non-smoking women aged 18 years or over by socio-demographic characteristics in Jilin Province, China. Passive smoking among non-smoking women was similar between Han (60.5%, 95% CI: 59.2–61.8) and Minorities (61.0%, 95% CI: 56.7–65.2). The majority of passive smokers were between 18–34 year olds, and the prevalence of passive smoking declined by age; 66.8 (61.6–71.6) from 18–24 year olds, 65.9 (62.9–68.7) from 25–34 year olds, 65.6 (63.6–67.6) from 35–44 year olds, 60.6 (58.7–62.5) from 45–54 year olds, 50.4 (47.9–52.8) from 55–64 year olds, and 38.9 (34.9–43.0) from 65–79 year olds. There were fewer passive smokers among those with lower education level (55.8%, 95% CI: 53.7–57.9), widowed (35.0%, 95% CI: 30.2–40.1), retired women (51.7%, 95% CI: 49.3–54.1) and among women whose family monthly income is more than 5000 RMB (53.4%, 95% CI: 45.3–61.4).

Table 4 describes the associations between socio-demographic factors and passive smoking by multivariable logistic regression. Participants residing in urban areas were less likely to passively smoke than those in rural (OR: 0.825, 95% CI: 0.729–0.935). Participants aged 45–79 years old were less likely to passively smoke compared to those aged 18–24 years old (45–54 years old (OR: 0.655, 95% CI: 0.472–0.910), 55–64 years old (OR: 0.481, 95% CI: 0.342–0.674), and 65–79 years old (OR: 0.351, 95% CI: 0.241–0.511)). Separated/Divorced (OR: 0.701, 95% CI: 0.500–0.982) and Widowed (OR: 0.564, 95% CI: 0.440–0.722) were less likely to passively smoke than married females. Retired women (OR: 0.810, 95% CI: 0.708–0.928) were also associated with a lower likelihood of passively smoking. Participants with a family monthly income of 5000 RMB and over (OR: 0.615, 95% CI: 0.432–0.876) were less likely to passive smoking than those with income of 500 RMB and less. Education level was not associated with passive smoking.

**Table 3:** Prevalence of passive smoking among non-smoking women aged 18 years or over by socio-demographic characteristics in Jilin Province, China.

Characteristic	Urban % (95% CI) (*n*)	Rural % (95% CI) (*n*)	Total % (95% CI) (*n*)
**Ethnic**			
Han	58.2 (56.6–59.9) (2731)	63.4 (61.5–65.4) (2542)	60.5 (59.2–61.8) (5273)
Minorities	59.4 (53.8–64.8) (245)	63.4 (56.3–70.0) (189)	61.0 (56.7–65.2) (434)
**Age**			
18–24	62.0 (55.3–68.2) (184)	72.4 (64.0–79.5) (117)	66.8 (61.6–71.6) (301)
25–34	65.1 (61.3–68.7) (477)	66.8 (62.2–71.1) (325)	65.9 (62.9–68.7) (802)
35–44	63.0 (60.3–65.7) (873)	69.0 (66.0–71.8) (745)	65.6 (63.6–67.6) (1618)
45–54	60.3 (57.6–63.0) (820)	61.0 (58.2–63.7) (817)	60.6 (58.7–62.5) (1637)
55–64	47.9 (44.3–51.5) (446)	53.6 (50.4–56.7) (566)	50.4 (47.9–52.8) (1012)
65–79	37.2 (31.6–43.1) (176)	41.7 (36.4–47.1) (161)	38.9 (34.9–43.0) (337)
**Education**			
Primary school and below	43.5 (39.4–47.6) (344)	60.4 (58.0–62.8) (1338)	55.8 (53.7–57.9) (1682)
Junior middle school	58.8 (55.8–61.8) (780)	66.6 (63.2–69.9) (826)	62.8 (60.5–65.1) (1606)
Senior middle school	60.0 (57.3–62.6) (1091)	62.3 (57.0–67.4) (345)	60.5 (58.1–62.8) (1436)
College and above	62.5 (59.1–65.7) (761)	66.7 (59.0–73.5) (222)	63.4 (60.3–66.4) (983)
**Marital status**			
Married	59.3 (57.6–60.9) (2528)	64.8 (63.0–66.7) (2556)	61.9 (60.6–63.1) (5084)
Single	63.3 (57.4–68.8) (269)	63.8 (51.8–74.2) (66)	63.4 (58.1–68.4) (335)
Separated/Divorced	46.9 (38.0–55.9) (58)	77.2 (59.2–88.8) (20)	52.6 (44.3–60.8) (78)
Widowed	36.5 (29.8–43.6) (121)	32.5 (26.7–38.8) (89)	35.0 (30.2–40.1) (210)
**Occupation**			
Manual	64.3 (61.7–66.8) (1100)	64.6 (62.4–66.8) (1839)	64.5 (62.8–66.1) (2939)
Skilled	64.0 (61.1–66.8) (910)	64.3 (58.5–69.7) (353)	64.1 (61.5–66.6) (1263)
Retired	48.2 (45.5–51.0) (966)	59.4 (54.9–63.7) (539)	51.7 (49.3–54.1) (1505)
**Family monthly income**			
<500	57.1 (52.1–62.0) (251)	60.6 (57.7–63.5) (903)	59.7 (57.2–62.2) (1154)
500–999	53.1 (49.1–57.1) (431)	64.4 (60.6–67.9) (698)	59.7 (56.9–62.4) (1129)
1000–1999	58.9 (56.4–61.3) (1204)	66.9 (62.9–70.6) (618)	61.5 (59.4–63.6) (1822)
2000–2999	58.9 (55.2–62.6) (614)	65.5 (59.3–71.1) (278)	60.8 (57.7–63.9) (892)
3000–4999	64.7 (59.7–69.5) (299)	69.8 (59.9–78.1) (98)	65.9 (61.5–70.2) (397)
≥5000	50.2 (41.2–59.2) (77)	65.7 (46.6–80.8) (30)	53.4 (45.3–61.4) (107)

Note: Complex weighted computation was used in the statistical analysis.

**Table 4:** Association between socio-demographic factors and passive smoking among non-smoking women aged 18 years or over, Jilin Province, China.

Characteristic	*p*	OR	95% CI
**Ethnic**			
Minorities		1	
Han	0.66	0.96	0.78–1.17
**Region**			
Rural		1	
Urban	<0.01	0.82	0.73–0.94
**Age**			
18–24		1	
25–34	0.08	0.75	0.54–1.03
35–44	0.12	0.77	0.55–1.07
45–54	<0.01	0.66	0.47–0.91
55–64	<0.01	0.48	0.34–0.67
65–79	<0.01	0.35	0.24–0.51
**Marital status**			
Married		1	
Single	0.379	0.87	0.63–1.19
Separated/Divorced	<0.03	0.70	0.50–0.93
Widowed	<0.01	0.56	0.44–0.72
**Occupation**			
Manual		1	
Skilled	0.85	0.98	0.83–1.17
Retired	<0.01	0.81	0.71–0.93
**Education**			
Primary school and below		1	
Junior middle school	0.13	1.12	0.97–1.31
Senior middle school	0.53	1.06	0.89–1.25
College and above	0.20	1.16	0.92–1.46
**Family monthly income**			
<500		1	
500–999	0.26	0.91	0.78–1.07
1000–1999	0.98	1.00	0.86–1.17
2000–2999	0.16	0.87	0.72–1.06
3000–4999	0.68	1.05	0.83–1.34
≥5000	<0.01	0.62	0.43–0.88

Notes: OR = odds ratio; CI = confidence interval; Complex weighted computation was used in the statistical analysis.

## 4. Discussion

In recent years, multiple epidemiological studies have been performed on smoking of males and whole populations, but there is little data on the status of passive smoking on non-smoking women in Jilin Province, China. Knowing the association factors of passive smoking can be useful in reducing the prevalence of passive smoking to protect women in Jilin Province, China. Our study was the first large population-based survey to investigate the prevalence and associated factors of passive smoking among non-smoking women in Jilin Province, China. The study indicated a high prevalence of passive smoking among non-smoking women in Jilin Province, even though it was lower than the rates from the China Global Adults Tobacco Survey in 2010 (GATS, 71.6%) [7]. There were also a high prevalence of passive smoking among non-smoking women at both home and work places, but it was lower than reported by 2010 GAST China (63.9% and 53.2%, respectively) [7]. We suggest that restaurants in China should be smoke-free, especially high-end and fast-food restaurants to reduce high SHS exposure there. Moreover, compared with restaurants, people spend more time at workplace or home. Thus, they have more exposure SHS opportunities at work and in their home compared to restaurants.

All of this might be due to tobacco control efforts and people paying more attention to smoking. The high prevalence of passive smoking among non-smoking women suggests that an urgent need to control tobacco for non-smoking women.

Multivariate logistic regression analysis showed the associations between socio-demographic factors and passive smoking. More than half of Han and Minorities women were passive smokers. The likelihood of being a passive smoker was not different between ethnic minorities and Han. Due to the differences of culture and lifestyles between Han and Minorities, there is a need for further investigation of this aspect. Women in rural areas were more likely to be passive smokers than those in urban areas, which is consistent with previous studies [17,20]. The possible explanations for the results are that rural area populations may lack information and knowledge about passive smoking, and a lower education level than urban women [5,13,15], and thus rural areas are the target market for much of the tobacco industry. Compared with rural areas, urban areas often participate in anti-smoking campaigns and receive tobacco control education. The prevalence among women aged between 55 and 64 has begun to decline and they are less likely to have the impact of passive smoking, as the prevalence of people who quit smoking between 55 and 64 years is rising. As people get older, they realize that smoking is a risk factor for a variety of chronic diseases and thus become more health consciousness and to promote better health, change poor lifestyle choices like smoking, which is consistent with previous studies [6,21]. There is a lower prevalence of passive smoking among women aged 65 year old and over in our study, which is consistent with previous studies [15,22,23]. Possible explanations for the finding include the elderly have more time and energy to consider their health with age and participate in anti-smoking campaigns. Separated/Divorced and Widowed women were found to passive smoke less than married or single women, which is consistent with results reported before [24,25]. This might be due to a majority of these women living alone and less likely to be exposed to smoke at home. Manual workers were more likely to be passive smokers than skilled laborers and retired women. The finding is consistent with previous studies [26]. This might be due to manual female workers having a low socioeconomic status and face more psychosocial and physical stressors. Prior studies [14,27] found that the higher an individual’s education and income were, the less likely they were to be a smoker; however, we found that the prevalence of passive smoking among women were all more than 50%. This might be because women who have a high socioeconomic status tend to also be married and therefore have a higher likelihood to be exposed to smoke.

The strength of the study lies in the large population-based representative sample survey. The limitation of the study is the properties of the cross-sectional study and the recall bias of all self-reported questionnaire interviews. Besides, the definitions and frequency of passive smoking were not measurements, and participants who were too weak or ill to complete the interviews were excluded.

## 5. Conclusions

The prevalence of passive smoking is lower than that reported in 2010 GATS China, but passive smoking is still prevalent and has been an acute public health problem among non-smoking women in Jilin Province, China. Our findings suggest an urgent need for tobacco control in Jilin Province, China and the efforts of public health authorities should be both comprehensive and focus on high-risk populations.
